# Students with learning disabilities/attention-deficit/hyperactivity disorder in higher education dealing with remote learning: lessons learned from COVID-19 era

**DOI:** 10.3389/fpsyg.2023.1172771

**Published:** 2023-05-12

**Authors:** Miriam Sarid, Orly Lipka

**Affiliations:** ^1^Department of Learning Disabilities and Education, Western Galilee College, Akko, Israel; ^2^Department of Learning Disabilities, Edmond J. Safra Brain Research Center for the Study of Learning Disabilities, University of Haifa, Haifa, Israel

**Keywords:** ADHD, adjustment, higher education, learning disabilities, satisfaction with life, Post-secondary education, COVID-19, Remote learning

## Abstract

**Introduction:**

The current study aimed to examine how students with learning disabilities (LD) and/or attention-deficit/hyperactivity disorder (ADHD) adjusted to higher education during the transition to remote learning (RL) in Israel during the COVID-19 pandemic.

**Methods:**

The study involved 621 undergraduate students, 330 of whom participated during the COVID-19 pandemic and 291 before the pandemic. Among these students, 198 had been diagnosed with LD and/or ADHD, while 423 had no reported disabilities (control group).

**Results:**

Students with LD/ADHD generally had lower adjustment scores during face-to-face learning and RL than the control group. In-depth analyses of four subgroups revealed that students with LD + ADHD reported lower academic, emotional, and institutional adjustments as well as reported lower satisfaction with life during RL than the control group members. ADHD was found to directly predict low satisfaction with life through the mediation of adjustment scores.

**Discussion:**

In conclusion, it is recommended that support be provided to high-risk LD/ADHD populations during a crisis. Furthermore, the implication of this study can inform intervention during emergency times.

## Introduction

Higher education is considered an important tool for improving social mobility and achieving an individual’s economic goals. Specifically, it predicts gainful employment and creates opportunities for meaningful occupations, career development, and quality of life (Sachs and Schreuer, 2011; Lipka et al., 2020).

Students with disabilities are a growing subpopulation at two-year and four-year post-secondary institutions (Newman et al., 2011). Among this subpopulation, students with learning disabilities (LD) and/or attention-deficit/hyperactivity disorder (ADHD) are the largest group (Cortiella and Horowitz, 2014). Although students with LD (60.9%) and students without disabilities (62.1%) have a similar ratio of enrolment in higher education institutions, students with LD have a low completion rate of 41%, whereas the general student population has a completion ratio of 52% (Lightfoot et al., 2018). Compared to students without disabilities, students with disabilities are more likely to attend a two-year community college or a vocational, technical, or business school than a four-year college (Joshi and Bouck, 2017). One of the challenges faced by students with LD/ADHD is adjusting to higher education (Likpa et al., 2020).

Most higher educational institutions worldwide, including those in Israel, switched to emergency remote learning (RL) because of the COVID-19 outbreak (Gewin, 2020). Prior to the COVID-19 outbreak, RL was perceived as a flexible and affordable means of complementing traditional higher education (Zhang et al., 2022). Zhang et al. (2022) presented a bibliometric review of over 1,000 articles on RL published since the COVID-19 outbreak, with a focus on the challenges of RL and how the situation affected universities and students. Their review refers to.

clusters of articles regarding factors such as student satisfaction, motivation, and self-efficacy. Undergraduate students without disabilities have been found to have less successful academic, emotional, social, and institutional adjustments to higher education during RL (Lipka and Sarid, 2022).

Despite the vast number of studies on RL in higher education institutions, there are only a few publications on the challenges faced by students with disabilities (Zhang et al., 2022) or their adjustment to higher education while using RL during the COVID-19 pandemic. The current study aims to fill the gap regarding how students with LD/ADHD adjusted to RL in higher education during the COVID-19 lockdown in comparison to students without LD/ADHD, as well as to compare both populations before the COVID-19 pandemic.

LD or ADHD students are often classified in research and clinical settings as one unified group of LD and/or ADHD (DuPaul et al., 2013), and ADHD is occasionally classified as a type of LD (Bizier et al., 2014). Since students with LD, ADHD, and LD + ADHD display unique differences in academic self-efficacy and perceptions of academic grades, Budd et al. (2016) suggest classifying them as separate groups both in research and clinical practice. Therefore, in addition to a general comparison of students with and without LD/ADHD, we examined how different subgroups of students with (i.e., LD, ADHD, and LD + ADHD) adjusted to higher education during RL.

## Theoretical background

### Students with LD

Studies have demonstrated that students with LD experience emotional and academic difficulties in academic settings, including lower self-worth (Shany et al., 2013) and a lower probability of graduating from college than their peers (Wagner et al., 2005; Advokat et al., 2011). The academic difficulties of students with LD are manifested as deficiencies in semantic fluency (Hall et al., 2017), note-taking, test readiness, listening comprehension, reading and writing skills, proper word identification, and sentence phrasing (Heiman and Precel, 2003; Skinner and Lindstrom, 2003; Heiman and Kariv, 2004; Vogel et al., 2007). Stothers and Klein (2010) discovered that students with LD have issues with processing speed, reading speed, decoding, and working memory. Callens et al. (2012) discovered that students with dyslexia were disadvantaged under time constraints and pervasively struggled with mathematical calculations. Lipka et al. (2020) compared undergraduate students with LD/ADHD to undergraduate students without LD/ADHD and discovered that the former had lower college admission grades than the latter. Students with LD who demonstrate low levels of adjustment to college have been reported to require additional counseling services and academic support (Saracoglu et al., 1989).

### Students with ADHD in higher education

The estimated prevalence of higher education students diagnosed with ADHD ranges from 2 to 8% (Garnier-Dykstra et al., 2010; Fleming and McMahon, 2012). Higher education students with ADHD are highly likely to have low grades, experience academic difficulties, withdraw from classes, and drop out of school (Advokat et al., 2011; Weyandt et al., 2013). Students with ADHD are also highly likely to experience psychological difficulties (Weyandt and DuPaul, 2006) and have been reported to have low levels of self-esteem and social skills, both of which are related to college adjustment (Shaw-Zirt et al., 2005). Unlike students with LD, students with ADHD have the option of receiving medications to address their difficulties. However, students tend to use stimulant medications to make up for poor study strategies rather than to reinforce good study habits (Advokat et al., 2011).

### Comorbid population with LD and ADHD

The comorbidity rate for students with LD + ADHD in the K-12 population is about 31–45% (DuPaul et al., 2013). Not much is known about this population of students in college, including their transition to college and the potential impact of their comorbidity on their functioning in higher education (DuPaul et al., 2017). However, in the younger population of students, comorbid individuals have been found to have more severe learning problems than students with only LD. Mayes et al. (2000) conducted a study on 119 children who were diagnosed with LD, ADHD, and LD + ADHD at the ages of 8–16 years and discovered that students with comorbid LD + ADHD had more severe attention problems than students with only ADHD. Based on their findings, they suggested that learning and attention problems are a continuum, making students with LD + ADHD the most severe case.

Students who have been diagnosed with ADHD are known to have a persistent pattern of inattention and/or hyperactivity/impulsivity that interferes with their functioning (American Psychiatric Association, 2013). College students who have ADHD may exhibit behaviors that interfere with their success and are related to low college GPAs; these behaviors include excessive procrastination, inadequate learning skills, and poor time management (Reaser et al., 2007; Advokat et al., 2011). Students with ADHD have been reported to be more forgetful and experience more difficulties completing assignments, sustaining attention, listening, and organizing tasks in post-secondary education than students without ADHD (Lewandowski et al., 2008). Students with ADHD have greater deficits in planning (Marzocchi et al., 2008), managing time, concentrating, selecting main ideas, and using study aids than students with only LD (Reaser et al., 2007). Budd et al. (2016) compared the achievements and academic self-efficacy of post-secondary students with LD, ADHD, and LD + ADHD and revealed that students with ADHD (with or without comorbidity) had lower grades and less course-related self-efficacy, such as time management and keeping up to date with schoolwork, than students with only LD. The authors highlighted the need to avoid the common practice of classifying students with ADHD and students with LD as one unified group, considering that students with only ADHD or LD and students with comorbidities have different profiles.

### Adjustment to higher education

A key determinant of student retention and success in post-secondary education is the ability to adjust to frequently complex environments. Students who struggle with post-secondary adjustments are highly likely to drop out (Adams and Proctor, 2010).

Baker and Siryk (1984) proposed a multifaceted view of adjustment, suggesting that academic adjustment comprises functioning in four distinct domains and that the quality of an adjustment can vary between domains. The first domain is ‘academic achievements.’ This domain includes motivation for learning, compatibility of skills with academic requirements, and the ability to earn satisfactory grades. The second domain, ‘social adjustment,’ relates to involvement in the study environment, including the ability to establish social networks. The third domain, ‘personal-emotional adjustment,’ reflects psychological and physical conditions; it is indicative of self-perception and represents the ability to handle study-related challenges that can cause stress and anxiety. The fourth and final domain, ‘institutional adjustment,’ involves students’ feelings about academia in general and their goal commitments (Baker and Siryk, 1984, 1989; Gerdes and Mallinckrodt, 1994).

### Adjustment to higher education among students with LD or ADHD

Upon transitioning from high school to higher education, students with disabilities face emotional challenges, such as anxiety and frustration regarding the academic requirements of the university environment, and social challenges associated with moving to a new and unknown place, such as feeling anonymous and learning alone (Hadley et al., 2003). Skinner (2004) interviewed 20 college graduates with LD to identify the variables that facilitate academic success. The main themes that emerged from the interviews were the importance of self-advocacy knowledge, the importance of accommodations and support systems, and the positive outcomes of goal setting.

Students with LD in post-secondary education face challenges that are similar to those faced by students without LD but, in addition, they face specific challenges related to their disabilities (Baker and Siryk, 1984). Undergraduate students with LD display lower levels of adjustment to college than their peers and express a substantial need for counseling services and academic support (Saracoglu et al., 1989). Studies that have compared students with disabilities to students without disabilities in terms of the four domains of academic, social, personal-emotional, and institutional adjustments have revealed that students with disabilities display poorer adjustments on the social, personal-emotional, and institutional adjustment subscales than students without disabilities (Adams and Proctor, 2010; Lipka et al., 2020). In particular, students with LD/ADHD have been reported to have lower adjustment scores and academic success rates than students without LD/ADHD (Krisher and Shechtman, 2016; Lipka et al., 2020).

The likelihood of ADHD students graduating is significantly lower than that of students without ADHD (Weyandt and DuPaul, 2006). Rabiner et al. (2008) explored adjustment to college among a large sample of students with self-reported ADHD in the US regarding personal, social, and academic aspects. They discovered that students with ADHD experience greater concerns about their academic performance and have higher depression scores than the general population.

Considering the difficulties encountered by many individuals with LD/ADHD, it is not surprising that they experience serious challenges in post-secondary education, which is a pivotal stage with life-changing effects (DuPaul et al., 2009). Students with LD/ADHD have been shown to struggle in their academic careers, achieving lower grades (Wagner et al., 2005; Jorgensen et al., 2009) and exhibiting higher academic procrastination levels (Hen and Goroshit, 2014), higher academic stress levels (Heiman, 2006), and less academic and social integration (DaDeppo, 2009) than their peers without LD/ADHD.

Students’ adjustment to post-secondary education can be related to stressful situations and life events (Solberg Nes et al., 2009). The transition to emergency RL during the COVID-19 outbreak was related to emotional outcomes, such as uncertainty about future events and anxiety (Sahu, 2020), as well as academic challenges associated with adapting to the transition from face-to-face learning to RL (Gewin, 2020). Therefore, it is instructive to examine how students who are highly likely to experience difficulties with RL adjust in comparison to their peers without LD/ADHD.

### Satisfaction with life

Satisfaction with life is a subjective assessment of personal well-being that reflects how people perceive their overall happiness and the quality of various aspects of their lives. Factors that determine satisfaction with life include employment, leisure, housing and living conditions, family and social ties, religion, and beliefs (Accordino and Herbert, 2000).

Veenhoven (1996) defined satisfaction with life as the product of evaluating one’s life, whereas Bradburn (1969) presented a broader view, which states that satisfaction with life includes two independent factors, namely positive and negative and that the balance between them determines happiness. Life satisfaction is believed to be partly determined by personality factors (Diener et al., 1999), but it can also be affected by a particular life domain when an individual actively pursues and advances toward personally valued goals (Brunstein, 1993; Lent et al., 2009). This study aims to compare how life satisfaction is affected by changes in learning set-ups and student adjustment to higher education.

Attention-deficit/hyperactivity disorder symptoms and certain ADHD-related behaviors (e.g., antisocial behaviors and an inability to positively engage and socialize with others) are negatively related to satisfaction with life. People with a history of ADHD symptoms are highly likely to experience repeated failures and difficulties that can undermine their general satisfaction with life (Young and Bramham, 2008). Rosenau et al. (2019) examined multiple epidemiological, health-behavioral, and psychological factors in post-secondary students and discovered that those diagnosed with ADHD had significantly low levels of overall well-being.

### The current study

Adjustment to higher education plays an important role in students’ retention rates (Tinto, 1993) and academic achievements (Brady-Amoon and Fuertes, 2011). Previous research on the population of students with LD/ADHD in face-to-face learning has shown that their adjustment to higher education is poorer than that of students without LD/ADHD (Lipka et al., 2020). Moreover, the transition to RL during the COVID-19 outbreak affected higher education students in various spheres of life (Zhang et al., 2020). Despite the significant impact of successful adjustment on educational outcomes, only a few studies have examined the aspects of adjustment among students with LD/ADHD during emergency RL in comparison to face-to-face learning and investigated the differences in adjustment between subgroups of students with disabilities (i.e., students with LD, ADHD, and LD + ADHD). Therefore, this study has three objectives. First, we aimed to compare how students from two groups: students with LD and/or ADHD and students without LD/ADHD adjusted to higher education during RL (during the COVID-19 pandemic) versus during face-to-face learning (before the COVID-19 pandemic) and their subjective well-being before COVID-19 and during COVID-19. Second, we aimed to examine how the adjustment of students from four groups: students with LD, students with ADHD, and students with LD + ADHD differed from that of students without LD/ADHD, all of whom studied during RL. Finally, we examined how students’ adjustment to RL in higher education affected their satisfaction with life.

## Methods

### Participants and data collection

This study involved overall 621 Hebrew-speaking undergraduate student participants from 30 universities and colleges in Israel.

#### Pre-COVID-19 sample

Prior to the pandemic, data was collected on a group of 291 undergraduate students as part of a larger dataset in a previous study (Lipka et al., 2020). This group provided a unique opportunity to be used as controls for comparison with undergraduate students who studied in higher education institutions in Israel during the COVID-19 outbreak. This group comprised 65 (22%) students with LD/ADHD and 226 students without LD/ADHD. In this sample, no data were collected regarding the participants’ ages.

#### COVID-19 sample

During COVID-19 (spring 2020) data were collected from 330 participants, including 133 (40%) with LD/ADHD and 197 students without LD/ADHD. Among these participants, 25 (7.6%) self-reported having LD only, 68 (20.6%) self-reported having ADHD, 40 (12%) self-reported having LD + ADHD, and 197 (59.7%) reported no LD/ADHD. Hundred and ninety three (58.5%) of the students (*n* = 330) responded to a questionnaire that was posted on social media, and 137 (41.5%) were recruited from an online panel (Panel4All). No difference was found between students with LD/ADHD and students without LD/ADHD with respect to gender, $x^{2}=4.67,\;\;p=.13$ and learning institution, $x^{2}=6.11,\;\;p=.11.$ Compared to students with LD/ADHD, students without LD/ADHD had a higher percentage of third-year students ($x^{2}=14.72,\;\;p<.001).$ All four groups had a mean age of 25 years (sd = 3.38), without significant differences between sub-groups. *F*(3,326) = 1.01, *p* = 0.39.

The ratio of LD/ADHD participants without LD/ADHD participants was significantly different between the COVID-19 and pre-COVID-19 samples $x^{2}=22.98,p<.001.$

Table 1 shows that the COVID-19 sample included fewer students (51%) from universities than the pre-COVID-19 sample ($x^{2}=9.85,\;\;p=.002$). Similar male/female ratios were found between the pre-COVID-19 (74% females) and COVID-19 (75% females) samples as well as between the LD/ADHD (77% female) and without LD/ADHD (73%) groups, $x^{2}=0.04,\;\;p=.85.$ The pre-COVID-19 sample included higher rate of students form third year of studies compare to COVID-19 sample, $x^{2}=94.98,p<.001.$ All participants enrolled in this study after being informed about its purposes and procedures and after signing an informed consent form.

**Table 1:** Demographic characteristics of the participants.

		Gender		Institution		Year of studying		
		Male	Female	College students	University students	First year	Second year	Third year
Pre-COVID-19 sample		76 (26%)	215 (74%)	106 (36%)	185 (63%)	17 (6%)	65 (22%)	209 (72%)
COVID-19 sample		84 (25%)	246 (75%)	161 (49%)	168 (51%)	102 (32%)	101 (32%)	115 (36%)
COVID-19 sample	Students with LD	3 (12%)	22 (88%)	16 (64%)	9 (36%)	6 (26%)	11 (48%)	6 (26%)
	Students with ADHD	18 (26%)	50 (74%)	34 (51%)	33 (49%)	22 (32%)	25 (37%)	21 (31%)
	Students with LD + ADHD	7 (18%)	33 (83%)	24 (60%)	16 (40%)	20 (53%)	10 (26%)	8 (21%)
	Students without LD/ADHD	56 (28%)	141 (72%)	87 (44%)	110 (56%)	54 (29%)	55 (29%)	80 (42%)

*p < .05, **p < .01, ***p < .001. LD/ADHD refers to undergraduate students with learning disabilities and/or attention-deficit/hyperactivity disorder.

It should be noted that since Israeli students obtain a BA degree over a period of 3 years, most of the pre-COVID participants were likely no longer BA students during COVID-19.

### Procedure

Electronic questionnaires were used to collect data for over 1 month during the COVID-19 pandemic in the spring of 2020 and during the year before the first wave of the pandemic.

#### Pre-COVID-19 sample

The participating academic institutions sent questionnaires to their students before the first wave of the pandemic. Social media posts were also used to invite interested students with LD, ADHD, or LD + ADHD to contact the research assistants. Students who were interested in participating were asked to provide a recent certification of diagnosis and to indicate the type of LD or ADHD they had.

Several institutions distributed the questionnaires through centers for students with disabilities. Electronic copies were sent to students with LD/ADHD at most higher learning institutions in Israel to capture a representative sample of students in the country. All students who expressed interest in participating were contacted via phone or email and informed about the research objectives, their importance, and the procedure. In addition, each participant’s LD/ADHD diagnosis (where relevant) and the institution of the study were confirmed by phone.

After providing verbal consent, the participants signed informed consent forms that confirmed their agreement to participate in this study and complete the online questionnaire. The questionnaire took approximately 40 min to complete. The students returned the questionnaires to the researcher via email and were offered $20 for their efforts.

#### COVID-19 sample

A portion of the sample (Table 1) was recruited through social media using a sampling and verification procedure similar to that of the pre-COVID-19 sample. The other portion of the sample was recruited through Panel4all. All underwent the same verification and consent procedures as the pre-COVID-19 sample. Each participant’s LD/ADHD diagnosis and the institution of the study were confirmed by phone. Data collection for students without LD/ADHD lasted a week. An extra 3 weeks were required to gather a sufficient number of participants with LD/ADHD.

The study was approved by the ethics committees of the University of Haifa, Faculty of Education, and Western Galilee College, in Israel.

### Instruments

#### Background and demographic questionnaire

The questionnaire included demographic information regarding gender, year of study, the institution of study, and self-report on the diagnosis of disability.

#### Student adaptation to college questionnaire

The original Student Adaptation to College Questionnaire (SACQ; Baker and Siryk, 1989) is a 67-item self-report questionnaire. It was developed in English and translated into Hebrew by Khalili (2006) using the standard back-translation method. The participants responded to each questionnaire item on a Likert scale ranging from 1 (does not apply to me at all) to 9 (applies to me very much), with relatively high scores representing enhanced adjustment. An abbreviated Hebrew version of the original SACQ (Baker and Siryk, 1989) had been developed earlier, with content adapted to the reality of Israeli university and college students (Lipka et al., 2020). To adapt the questionnaire to the COVID-19 pandemic and lockdown, we omitted items irrelevant to remote learning, such as meeting with other students. The adapted questionnaire consisted of four subscales: academic adjustment, which included 22 items addressing adaptation to academic challenges in the learning environment (e.g., ‘I find academic studies difficult’) and had a high internal consistency of Cronbach’s alpha = 0.87; personal-emotional adjustment, which included 10 items addressing general psychological distress (e.g., ‘I have been feeling tense or nervous lately’) and had a Cronbach’s alpha of 0.86; social adjustment, which included 7 items addressing the ability to cope with social interactions (e.g., ‘I am meeting as many people and making as many friends as I would like to in college’) and had a Cronbach’s alpha of .69; and institutional adjustment, which included 5 items addressing commitment to academic goals (e.g., ‘I am happy with my decision to attend this college’) and had a Cronbach’s alpha of .73. The institutional adjustment subscale also included items addressing the student’s intention to leave the academic institution or drop out (e.g., ‘Recently, I have been thinking a lot about quitting university/college’).

### Satisfaction with life scale

This scale (Diener et al., 1985, translated by Shrira and Shmotkin, 2008) is a widely used five-item index designed to measure global cognitive judgments regarding life satisfaction (well-being), which is associated with positive life outcomes (Lyubomirsky et al., 2005). The scale includes items such as ‘So far, I have achieved the important things I want in life’. Participants indicate the extent to which they agree or disagree with each of the five items using a scale ranging from 1 (strongly disagree) to 7 (strongly agree). The reliability of the translated questionnaire was .82. In this study, the internal reliability of the entire questionnaire was .85.

### Data analysis

The differences between students with LD/ADHD and students without LD/ADHD and the differences between the pre-COVID-19 sample and the COVID-19 LD/ADHD sample were evaluated using a two-way multivariate analysis of variance (MANOVA). The four adjustment types and satisfaction with life served as dependent variables.

A one-way MANOVA was conducted to examine the differences in adjustment and satisfaction with life between the four groups of students (i.e., students with LD, ADHD, LD + ADHD, and without LD/ADHD). The analysis was followed by *post hoc* testing (Tukey) for pairwise comparisons between the groups. Hierarchical linear regression was conducted to examine how LD and ADHD status, demographic characteristics, and adjustment subscales contributed to predicting satisfaction with life. Additionally, we used PROCESS Macro for SPSS (Hayes, 2009) to conduct a mediation analysis using satisfaction with life as the dependent variable, adjustment to higher education as the mediator, and ADHD as the independent variable. The bootstrap method was used in the analysis to validate the mediation effect. The analyses were conducted using SPSS for Windows version 29.

## Results

### Differences between students with LD/ADHD and without LD/ADHD assessed before COVID-19 and during COVID-19

The comparison between two groups of students: students with LD/ADHD and students without LD/ADHD during RL and face-to-face learning revealed that both groups of students demonstrated lower adjustment to higher education during RL than during face-to-face learning in the total score of adjustment, *F*(1,617) = 29.64, *p* < 0.001, *η_p_^2^* = 0.05 and observed statistical power value of 1, and subscales of adjustment, multivariate Wilk’s *F*(5,613) = 4.61, *p* < .001, η_p_^2^ = .04 and observed power of .97; (see Table 2 for means and univariate *F* values). The univariate comparisons revealed a decrease in total adjustment and its subscales and in satisfaction with life.

**Table 2:** Means, standard deviations, and univariate *F-*values for the groups’ adjustment to higher education and satisfaction with life of pre COVID-19 sample (face-to-face learning) and COVID-19 sample (RL).

Learning mode	Group		Total adjustment	Academic	Emotional	Social	Institutional	Satisfaction with life
Pre COVID-19 sample	Students without LD/ADHD	*M* (SD)	6.69 (0.93)	6.43 (1.08)	6.34 (1.49)	6.45 (1.13)	8.27 (0.88)	5.20 (1.06)
	Students with LD/ADHD	*M* (SD)	5.97 (0.97)	5.78 (1.08)	5.06 (1.73)	5.88 (1.42)	7.82 (1.30)	4.98 (1.12)
	All students	*M* (SD)	6.53 (1.01)	6.28 (1.11)	6.06 (1.64)	6.32 (1.22)	8.17 (1.00)	5.15 (1.08)
COVID-19 sample	Students without LD/ADHD	*M* (SD)	6.21 (1.06)	6.04 (1.17)	5.88 (1.51)	6.03 (1.25)	7.93 (1.37)	5.02 (1.10)
	Students with LD/ ADHD	*M* (SD)	5.44 (1.06)	5.42 (1.13)	4.57 (1.59)	5.45 (1.46)	7.28 (1.81)	4.48 (1.37)
	All students	*M* (SD)	5.90 (1.12)	5.79 (1.19)	5.35 (1.67)	5.79 (1.37)	7.67 (1.59)	4.80 (1.24)
Total	Students without LD/ADHD	*M* (SD)	6.47 (1.02)	6.24 (1.14)	6.13 (1.52)	6.25 (1.20)	8.11 (1.15)	5.11 (1.08)
	Students with LD/ADHD	*M* (SD)	5.62 (1.09)	5.54 (1.12)	4.73 (1.65)	5.59 (1.46)	7.46 (1.68)	4.64 (1.31)
	All students	*M* (SD)	6.19 (1.11)	6.02 (1.18)	5.68 (1.69)	6.04 (1.32)	7.90 (1.37)	4.96 (1.18)
*F* (eta^2^) df = 1,617	Learning mode		29.64*** (0.05)	13.94*** (0.02)	11.69*** (0.02)	13.88*** (0.02)	13.51*** (0.02)	10.55*** (0.02)
	Group		66.40*** (0.10)	39.11*** (0.06)	86.51*** (0.12)	25.02*** (0.04)	21.33*** (0.03)	13.62*** (0.02)
	Learning mode × group (interaction)		0.09 (0)	0.02 (0)	0.01 (0)	0.01 (0)	0.73 (0)	2.44 (0)

***p < .001.

Additionally, students with LD/ADHD from both samples (i.e., RL and face-to-face learning) had lower adjustment scores than students without LD/ADHD in total adjustment, *F*(1,617) = 66.40, *p* < .001, *η^2^* = .10 and observed statistical power value of 1, and the subscales of adjustment, multivariate Wilk’s *F*(5,613) = 18.60, *p* < .001, *η^2^* = .13 with observed statistical power value of 1. This result, when observed with no significant interaction effect of the group (students with LD/ADHD vs. students without LD/ADHD) and type of learning (during COVID-19 - RL, vs. pre-COVID-19 - face-to-face learning), revealed that all the students reported on decreased adjustment scores during COVID-19 (RL), whether or not they had an LD: Wilks’s *F*(5,613) = .84 and *p* = .52 for the interaction effect.

**Figure 1:**
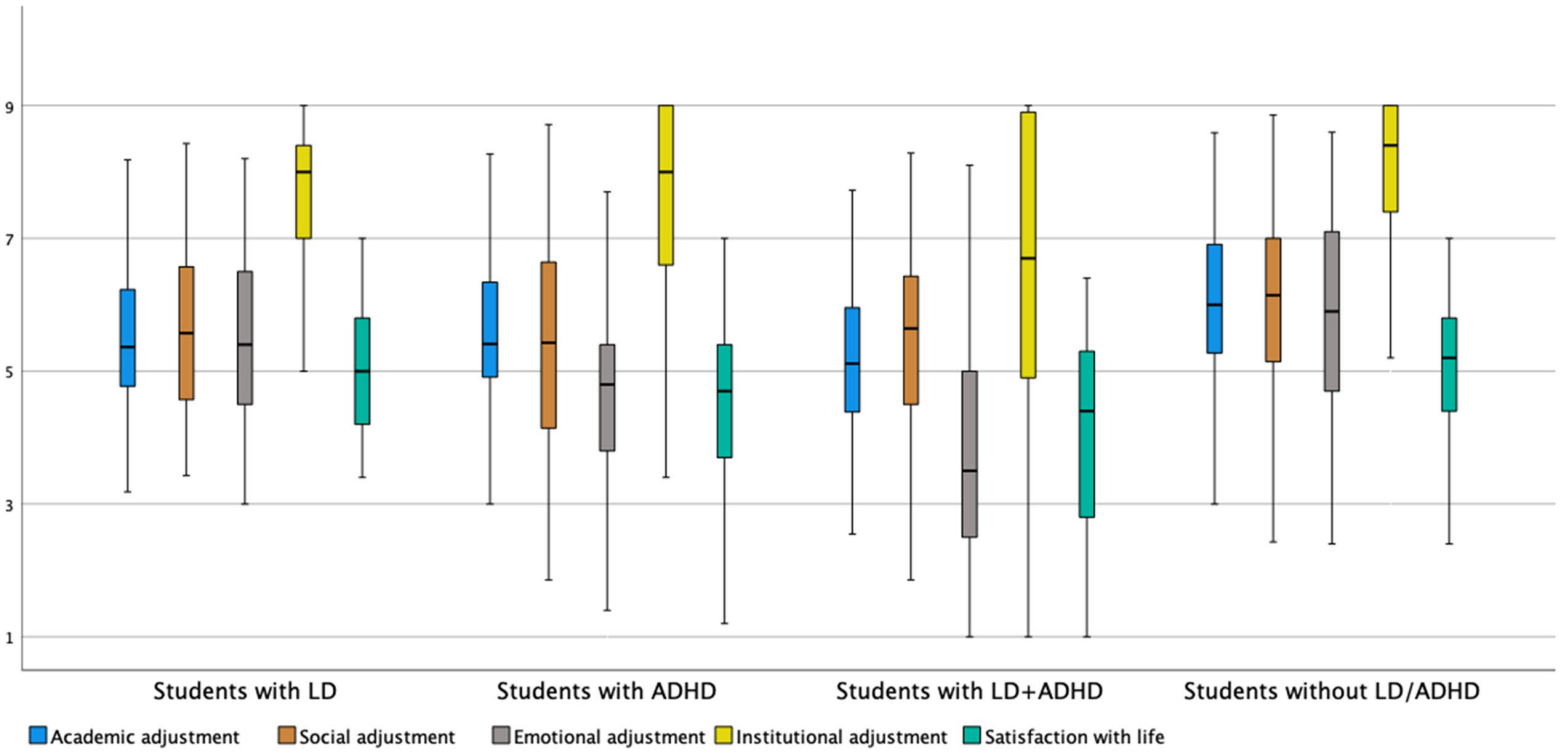
A boxplot of adjustment to higher education among COVID-19 sample (RL) and satisfaction with life of the groups (*n* = 330). Mean score in the y axis.**p < 0.01.

The second aim of the study was to examine the adjustment of four subgroups (i.e., students with LD, ADHD, LD + ADHD, and no LD/ADHD) according to their disabilities during COVID-19 (RL). The analysis was conducted for only the 330 students since we only had self-reports on the types of disabilities of the students who were learning remotely. The multivariate analysis revealed significant differences between the four groups of students with disabilities, in total adjustment score, *F*(3,327) = 17.14, *p* < .001, and η_p_^2^ = .14, and in its subscales, Wilk’s *F*(15,889) = 5.83, *p* < .001, and η_p_^2^ = .08 (see Table 3 for means and SDs and Figure 1).

**Table 3:** Means, standard deviations, and univariate *F-*values for adjustment to higher education and satisfaction with life by group (COVID-19 sample, RL).

Type of adjustment		**A**	**B**	**C**	**D**	*F*(*η^2^*) df = 3,326	*Post hoc* (Tukey)
		Students without LD/ADHD	Students with LD	Students with ADHD	Students with LD + ADHD		
Total Adjustment	*M* (SD)	6.21 (1.06)	5.79 (1.08)	5.53 (0.96)	5.07 (1.13)	17.14*** (0.14)	C, D < A; D < B
Academic	*M* (SD)	6.04 (1.17)	5.57 (1.21)	5.55 (1.04)	5.11 (1.17)	9.08***(0.08)	C, D < A
Emotional	*M* (SD)	5.88 (1.51)	5.55 (1.28)	4.58 (1.39)	3.95 (1.80)	25.60*** (0.19)	C, D < A, B
Social	*M* (SD)	6.03 (1.25)	5.60 (1.53)	5.41 (1.49)	5.41 (1.39)	5.12*** (0.05)	C, D < A
Institutional	*M* (SD)	7.93 (1.37)	7.52 (1.29)	7.53 (1.65)	6.70 (2.22)	7.41*** (0.06)	D < A, C
Satisfaction with life	*M* (SD)	5.02 (1.10)	5.07 (1.03)	4.50 (1.37)	4.06 (1.44)	9.11*** (0.08)	C, D < A; D < B

***p < .001.

*Post hoc* (Tukey) comparisons showed that students with ADHD or LD + ADHD scored lower on total adjustment than students without LD/ADHD, and students with LD + ADHD scored on total adjustment lower than students with only LD. The univariate and *post hoc* (Tukey) analyses showed that both groups of students with ADHD (i.e., students with ADHD and LD + ADHD) had lower academic and emotional adjustment scores than students without LD/ADHD. Students with ADHD and LD + ADHD also had lower emotional adjustment scores than students with LD and students without LD/ADHD. In addition, students with LD + ADHD were found to have lower institutional adjustment scores than all the other groups. Students with LD + ADHD also demonstrated lower satisfaction with life than students with LD and students without LD/ADHD.

Our third aim was to examine the unique contribution of adjustment to higher education to the explained variance of satisfaction with life during COVID-19 (RL). Table 4 presents the results. A hierarchical linear regression model was developed in two steps. In the first step demographic characteristics (i.e., gender, year of study in the college/university, age, and LD or ADHD as dummy variables) were included to control their possible covariate effect. In the second step, four adjustment subscales were entered into the model. The intercorrelations between the adjustment subscales varied from *r* = 0.46 to *r* = 0.64, and none of the VIF values reached 10, demonstrating that the multicollinearity assumption was not violated (Myers and Myers, 1990).

**Table 4:** Hierarchical regression analysis for predicting satisfaction with life using adjustment and demographic characteristics as predictors (COVID-19 sample, RL).

Model	First step		Second step	
	Beta	*p*	Beta	*p*
ADHD	−0.26	<0.001	−0.04	0.39
LD	−0.06	0.3	0.01	0.78
Age	−0.11	0.06	−0.04	0.40
First year	−0.02	0.73	0.08	0.14
Second year	−0.02	0.72	0.06	0.23
Gender (Female coded as 1)	−0.04	0.46	−0.01	0.85
Type of learning institute (University coded as 1)	−0.14	0.012	−0.06	0.23
Academic adjustment			−0.03	0.68
Emotional adjustment			0.43	<0.001
Social adjustment			0.24	<0.001
Institutional adjustment			0.12	0.039
Rsq (cumulative)	.10***		.39***	

***p < .001; the variables ADHD, LD, gender, first year, second year, and type of learning institute are coded as 0/1 dummy variables.

The results revealed a significant predictive model accounting for 39% of the variance of satisfaction with life. The first step of the model revealed that students with ADHD exhibited relatively low satisfaction with life and that students who were studying at a college rather than a university had relatively high satisfaction with life. These two variables accounted for 10% of the explained variance of satisfaction with life. Regarding adjustment to higher education, the social, emotional, and institutional adjustment scores increased the explained variance of satisfaction with life by 20%. In the second step, ADHD and type of learning institute were insignificant, probably due to covariability with the adjustment scales. Therefore, an additional mediation model was run (Hayes, 2009). It should be noted that based on the regression analysis that revealed a non-significant effect of the academic adjustment on satisfaction with life, the mediation model was conducted with the three significant adjustment subscales (i.e., emotional, social, and institutional). As each of the three subscales of adjustment had a significant contribution to the prediction of satisfaction with life, they were each included separately as mediators in mediation analysis.

### Mediation analysis

The obtained results revealed that having ADHD has an indirect effect on satisfaction with life. Students with ADHD reported having poor adjustments to higher education, and students who struggled to adjust were less satisfied with life. The three adjustment mediators had significant indirect effects.

**Figure 2:**
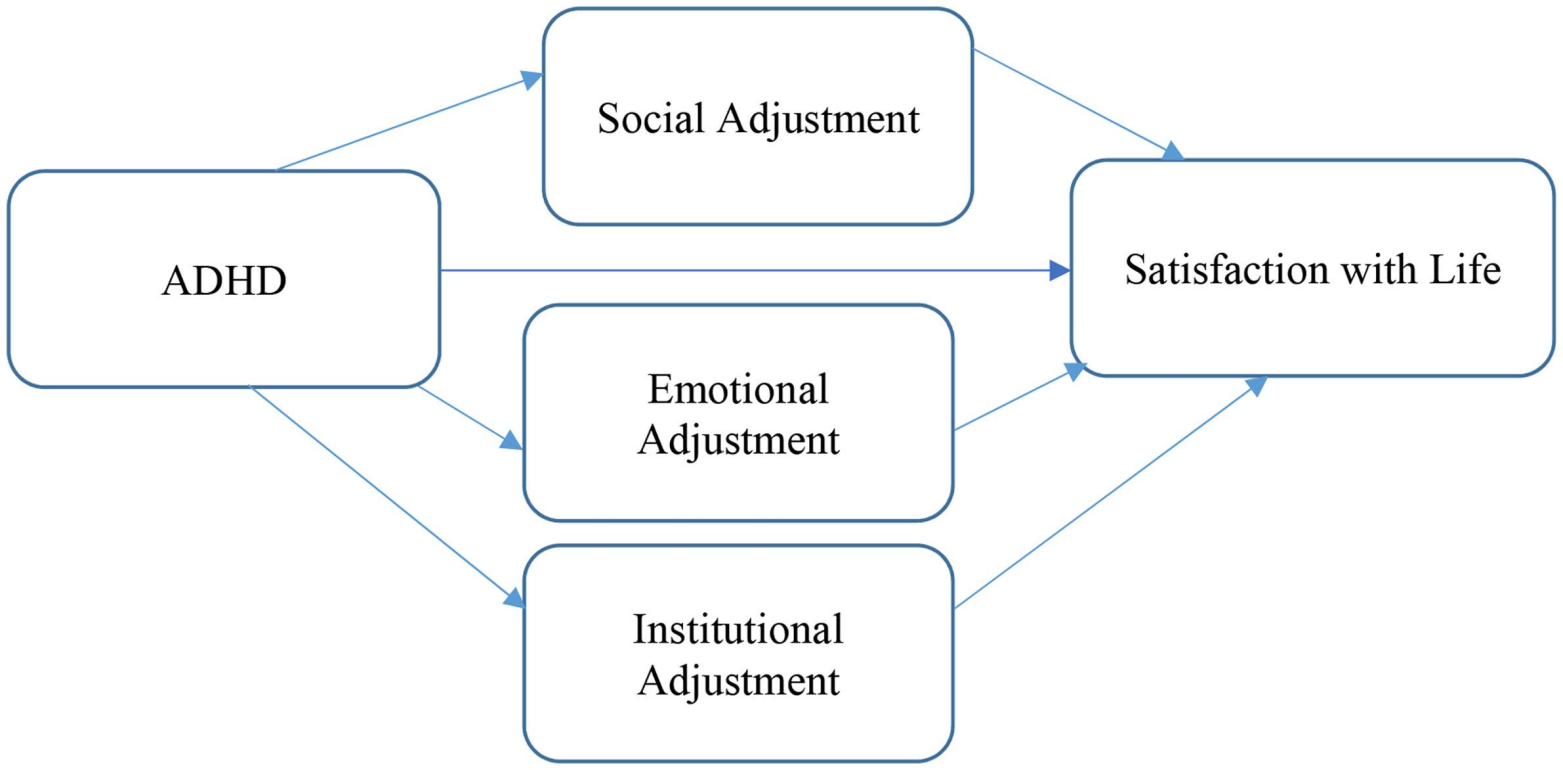
Mediation model for adjustment to higher education as a mediating factor in the relationship between ADHD and undergraduate students’ satisfaction with life.

The results are presented in Figure 2 and Table 5.

**Table 5:** Mediation analysis results for predicting satisfaction with life by ADHD and adjustment to higher education (COVID-19 sample, RL).

	*ß*	*t*	*SE*	LLCI	ULCI
Total effect	−0.69	−4.87**	0.14	−0.97	−0.41
Direct effect	−0.06	0.13	−0.5	−0.32	0.19
Indirect effects					
Emotional adjustment	−0.44		0.08	−0.61	−0.30
Social adjustment	−0.13		0.05	−0.23	−0.05
Institutional adjustment	−0.06		0.04	−0.15	−0.01

SE represents the standard error of ß; LLCI and ULCI represent the lower and upper confidence intervals for *t*. **p < .01.

## Discussion

The transition of higher education institutions to RL during the COVID-19 pandemic compelled students with LD/ADHD to adjust to an unfamiliar situation in the academic environment. This study aimed to examine the adjustment of students with LD/ADHD in light of RL. Specifically, we compared students with LD/ADHD to students without LD/ADHD in terms of multifaceted adjustment to higher education and well-being and its predictors during RL in the COVID-19 pandemic era, based on the assumption that RL is expected to continue in the post-pandemic era (Dwivedi et al., 2020; Conrad et al., 2022).

Overall, students assessed during the COVID-19 outbreak reported lower scores on overall adjustment and all adjustment measures than those assessed before the pandemic. This finding probably reflects rapid and significant changes in all aspects of undergraduate life during the pandemic which was an emergency time, and it aligns with previous findings that have been observed in the general population of students (Lipka and Sarid, 2022). Changes in the instruction method may have directly affected the self-reported academic adjustment scores, but the self-reported social and emotional adjustment scores were relatively low during the pandemic, presumably due to the prolonged absence of campus life. The decrease of self-reported adjustment may be attributed to the sudden change in the learning environment, academic requirements, and challenges such as distractions, reduced focus, increased workload, and technological difficulties that all students face during RL (Goegan et al., 2022; Lipka and Sarid, 2022). In addition to the high levels of stress and anxiety that were documented in the research during the pandemic (Lipka and Sarid, 2022; Zhang et al., 2020), the change in the learning environment may affect other aspects of students’ lives, such as the academic, emotional, social, and institutional aspects.

Satisfaction with life is defined as a measure of well-being, and it is sensitive to life events (Kobau et al., 2010). The reported decrease in this measure may also indicate that COVID-19 was viewed as a major life event with substantial effects on the overall quality of life. Gonzalez et al. (2020) recently examined anxiety among young adults in Spain and revealed their emotional response to COVID-19. They discovered that anxiety levels were higher during the pandemic than before it.

Furthermore, the study addresses the differences in adjustment and satisfaction with life between students with LD/ADHD and students without LD/ADHD before and during the COVID-19 outbreak. Since post-secondary students with LD/ADHD are at an increased risk of facing difficulties, we expected that students with LD/ADHD would report experiencing lower adjustment and satisfaction with life than their peers without LD/ADHD. This hypothesis was confirmed by all the adjustment measures (academic, social, emotional, and institutional) and satisfaction with life in both the pre-COVID-19 and COVID-19 samples. The differences between the groups are consistent with previous findings on the academic and psychosocial states of undergraduates with LD/ADHD (DuPaul et al., 2017) and a recent finding on students with and without LD/ADHD before the pandemic (Lipka et al., 2020). Since adjustment appears to affect higher education outcomes, including achievements and attrition (Weyandt and DuPaul, 2006), students with LD/ADHD are highly likely to drop out, making the challenge of adjustment more salient for them than for other students. The findings of this study are supported by a recent finding on students with and without dyslexia in Poland (Zawadka et al., 2021). This recent finding revealed that students with reading difficulties failed exams more frequently at university, had lower academic achievements, experienced higher stress levels, and believed that more work was expected of them in RL than students without LD/ADHD. The difficulties that students with LD encounter in RL necessitate the provision of additional academic and emotional support during the transition to RL.

A recent study on the self-regulation learning of K-12 students highlighted the role of instructors as co-regulators in face-to-face learning and discovered that this support decreased under RL conditions (Carter et al., 2020). The authors described strategies that have emerged from previous research on self-regulation learning in online K-12 environments, including (a) asking students to consider how they learn online, (b) providing pacing support, (c) monitoring engagement with instructional materials, and (d) supporting families. Such strategies are likely to be beneficial in post-secondary institutions, possibly through self-regulation workshops for both students and instructors.

Our second goal was to examine the differences in the total adjustment score and adjustment subscores, and satisfaction with life between the three groups of students with LD, ADHD, and LD + ADHD, and students without LD/ADHD. In terms of adjustment to higher education, students with ADHD or comorbid LD + ADHD obtained relatively low total adjustment and in academic, emotional, and social adjustment scores on the SACQ. These findings are also supported by previous findings that revealed that freshman students with ADHD have greater academic concerns and more depressive symptoms in their transition to college than students without LD/ADHD (Rabiner et al. (2008)). A study that assessed the adjustment of students with self-reported ADHD (with or without LD) also indicated that they had lower adjustment scores than students without LD/ADHD (Shaw-Zirt et al., 2005). Therefore, the current results are consistent with previous reports stating that ADHD is associated with a low grade-point average, increased academic difficulties, and ineffective study skills (Advokat et al., 2011; Weyandt and DuPaul, 2013; DuPaul et al., 2017). Our results also support Tinto’s (1975) model and the proposition that student trajectories and academic persistence are shaped by high school and college experiences (Tinto, 2010). Students with LD/ADHD are likely to experience academic difficulties during their earlier school years, which might influence their academic adjustment during post-secondary education. These results indicate that students with ADHD, whether comorbid or without LD, function below normative adjustment in college/university regardless of whether face-to-face learning or RL is used, making them highly likely to drop out of school or experience academic failure.

Adjustment to higher education was identified as a mediating factor in the relationship between ADHD and satisfaction with life. Students who reported having ADHD experienced poor emotional and social adjustments to college/university. Previous research revealed that ADHD in adulthood may be related to mental disorders, such as depression and anxiety, and problems in one’s vocational life, such as unemployment or dropping out of school (Hennig et al., 2017). Therefore, it is not surprising that individuals with ADHD are less satisfied with their lives. The mediating effect of a multifaceted adjustment to higher education demonstrates that satisfaction with life is mediated by various aspects of students’ academic lives. The poor adjustment of ADHD students to higher education may be a reflection of some core ADHD symptoms and related problems, such as lack of emotional control, antisocial behavior, and failure to positively engage and socialize with others (Young and Gudjonsson, 2008). Consequently, this poorer adjustment is related to a low evaluation of the student’s personal quality of life. Some of the emotional and social problems that people with ADHD may face in ordinary times intensified during the emergence of RL. However, by understanding the relationship between ADHD, adjustment, and satisfaction with life, higher education institutions may implement preventive measures that will improve the adjustment of students with ADHD, which will consequently improve their satisfaction with life.

### Limitations

This study compared how students with LD/ADHD and students without LD/ADHD adjusted to higher education and were satisfied with life during RL and face-to-face learning. In this study, we did not ask the student to report the specific subtype of LD, as it was beyond the scope of the study. However, a further study that will inquire about the adjustment of various profiles of LD’ may put light on this topic.

In both phases of this study, we measured LD/ADHD based on students’ self-reports because we had no means of verifying the accuracy of this information. Although the students were asked to report if they had been diagnosed, some of them may have been misdiagnosed. Due to the fact that this study was based on self-reports, some students may have had difficulties disclosing their LD/ADHD diagnoses. Additionally, the adjustment measures used in this study were based on self-report questionnaires, which reflected the respondents’ subjective experiences. A subsequent study would supplement the subjective measures used here with objective adjustment measures, such as academic achievement.

### Implications for practice and research

The transition to RL during the COVID-19 pandemic created a unique situation that compelled students to cope with various types of stress (emotional, health, financial, and social) during a rapid shift in instructional methods. The findings of this study have several implications for practitioners working with undergraduate students with LD/ADHD. First, special attention should be paid to this population, which appears to be even more vulnerable to pandemic-related changes than their peers without LD/ADHD and is therefore more likely to require academic, social, and emotional support. Second, students with LD/ADHD should be encouraged to work on their self-regulation skills and concentration capacity to improve the effectiveness of RL.

In this study, students with ADHD were found to have more adjustment difficulties than other students. Therefore, their challenges should be considered by providing suitable support services at colleges or universities that can enhance the emotional, social, and institutional aspects of adjustment in addition to their academic challenges.

Higher education institutions will probably normalize partial or full RL in the post-pandemic era. Therefore, these institutions should be aware of the challenges that RL may pose to vulnerable populations, such as students with LD or ADHD, and provide them with workshops and the required support.

## Data availability statement

The original contributions presented in the study are included in the article/supplementary material, further inquiries can be directed to the corresponding author.

## Ethics statement

The studies involving human participants were reviewed and approved by the ethics committee of the university of Haifa, Department of Learning Disabilities, and the ethics committee of the Western Galilee College. The patients/participants provided their written informed consent to participate in this study.

## Author contributions

All authors listed have made a substantial, direct, and intellectual contribution to the work and approved it for publication.

## Funding

This study was sponsored by a grant from the Israeli National Insurance Institute and from the Edmond J. Safra Brain Research Center for the Study of Learning Disabilities, Haifa University, Haifa, Israel.

## Conflict of interest

The authors declare that the research was conducted in the absence of any commercial or financial relationships that could be construed as a potential conflict of interest.

## Publisher’s note

All claims expressed in this article are solely those of the authors and do not necessarily represent those of their affiliated organizations, or those of the publisher, the editors and the reviewers. Any product that may be evaluated in this article, or claim that may be made by its manufacturer, is not guaranteed or endorsed by the publisher.
